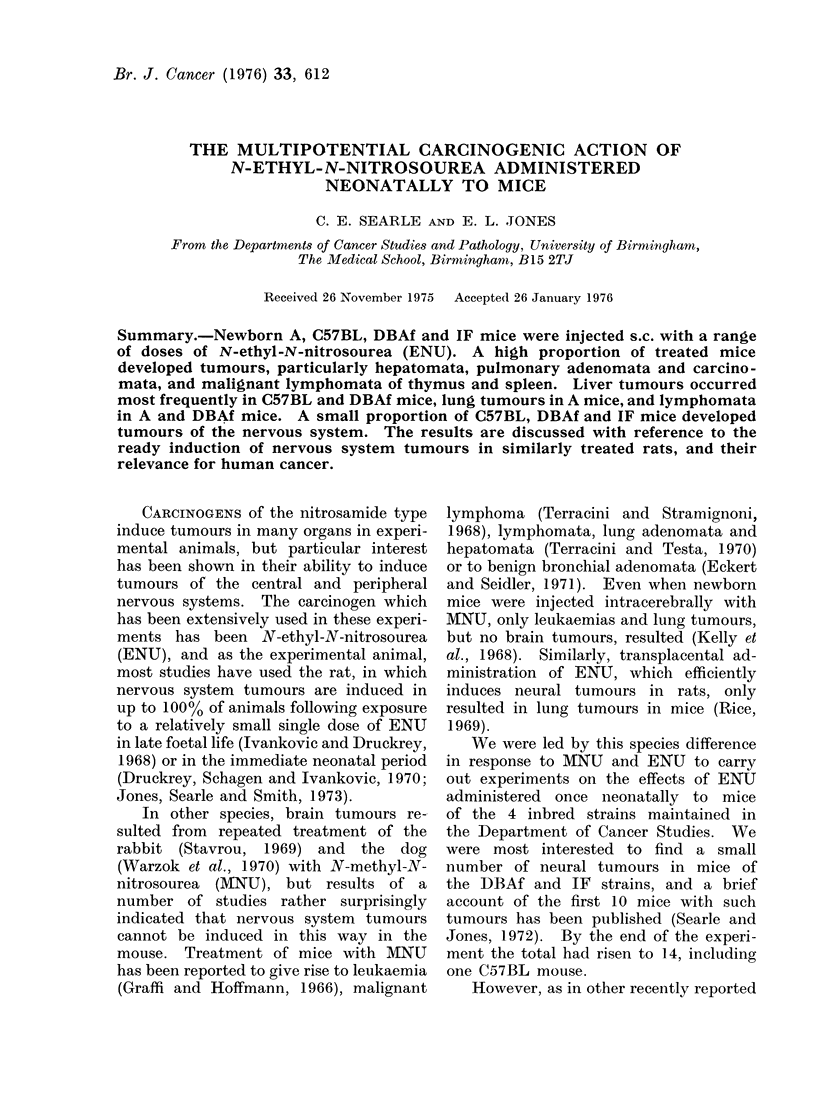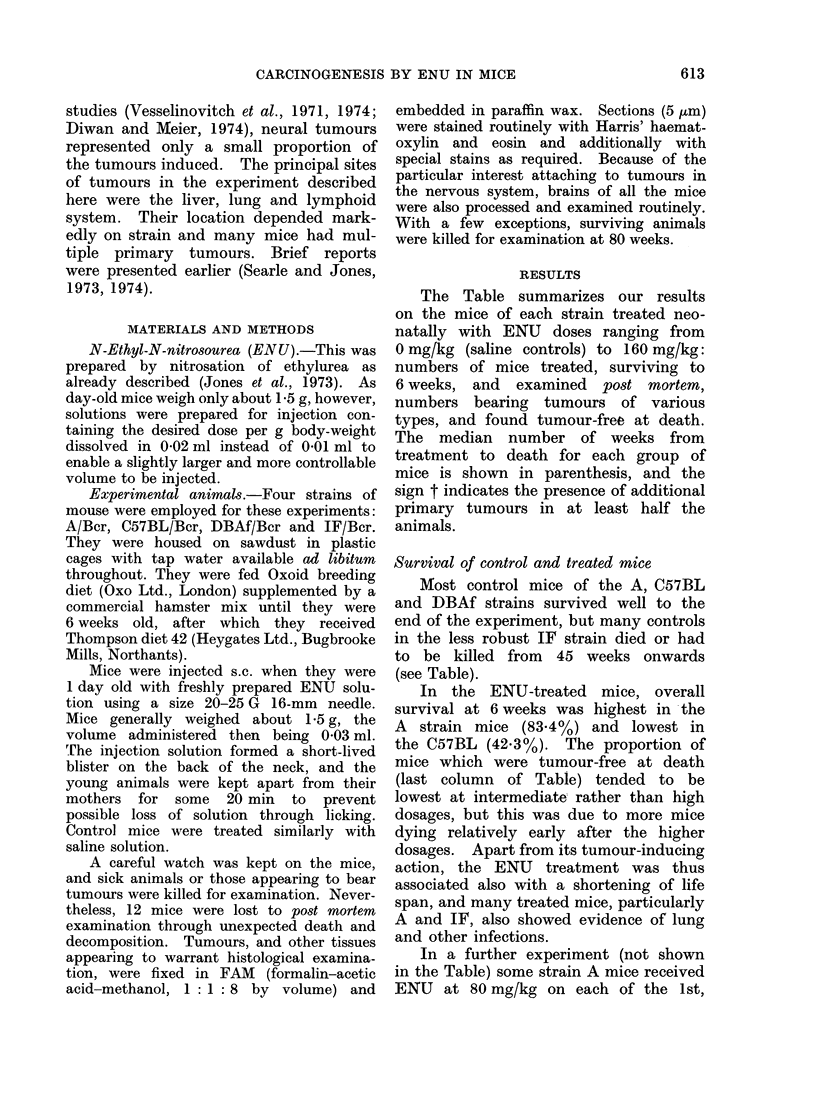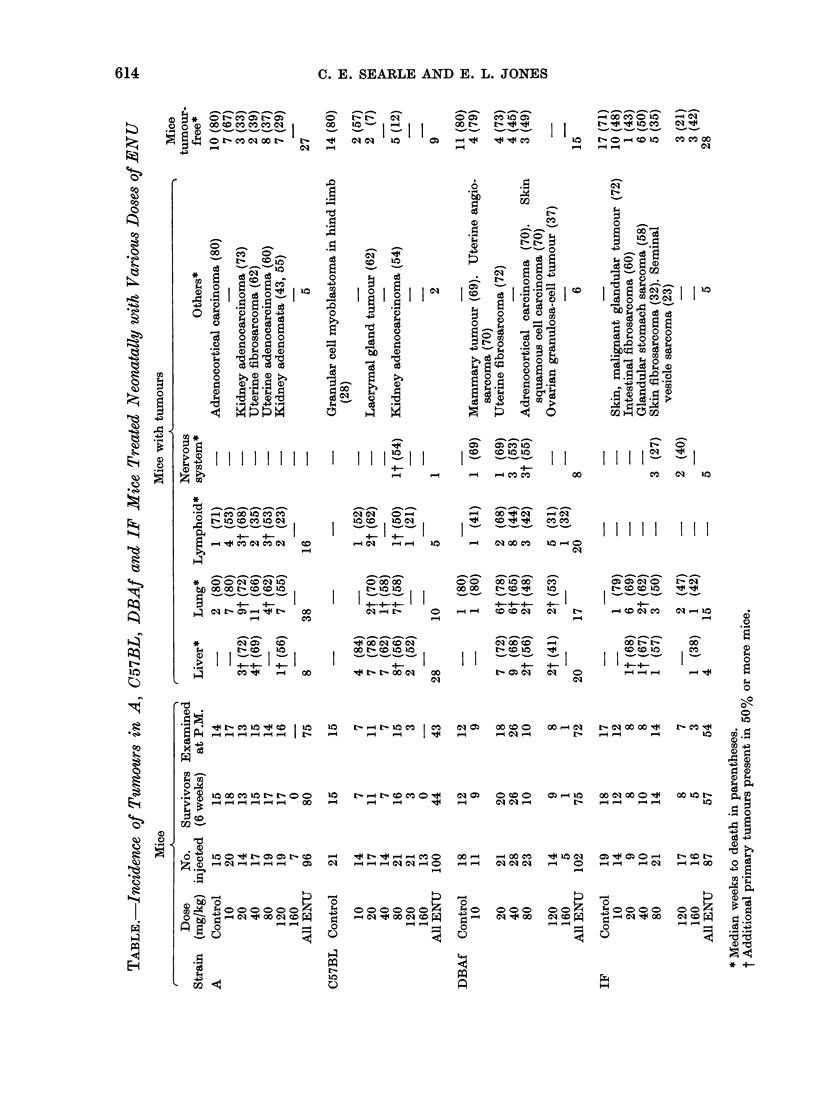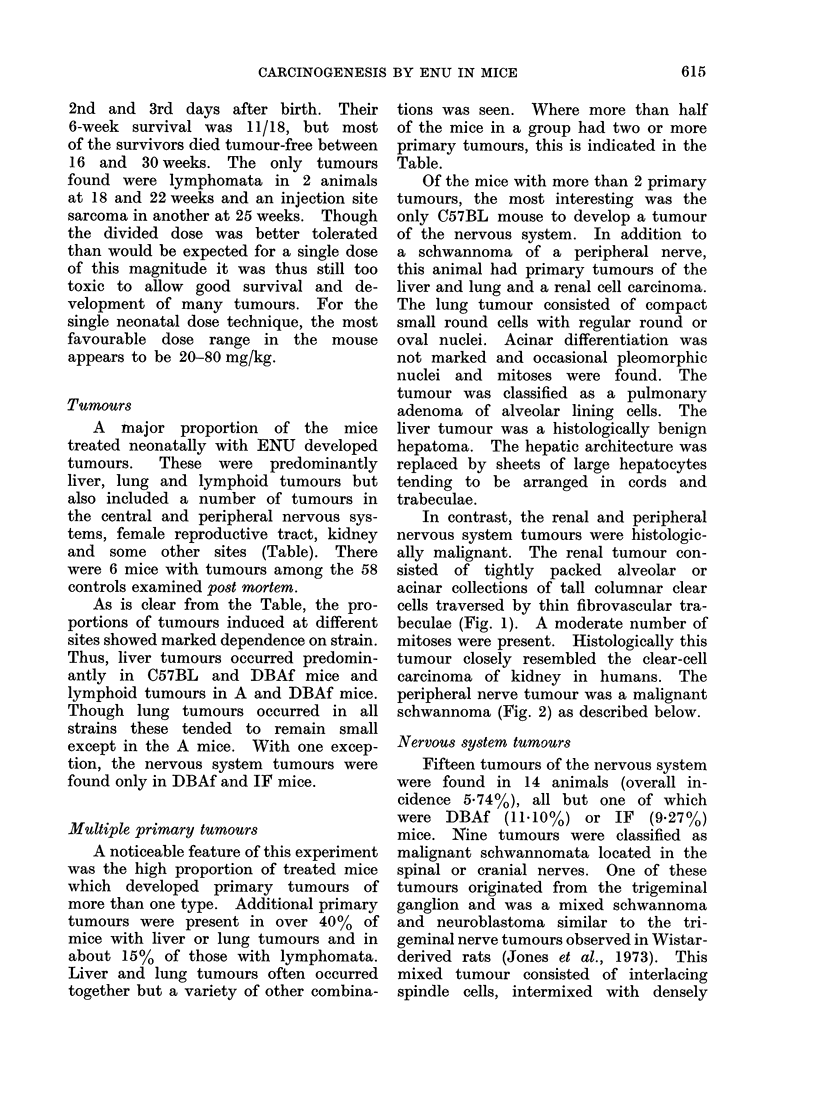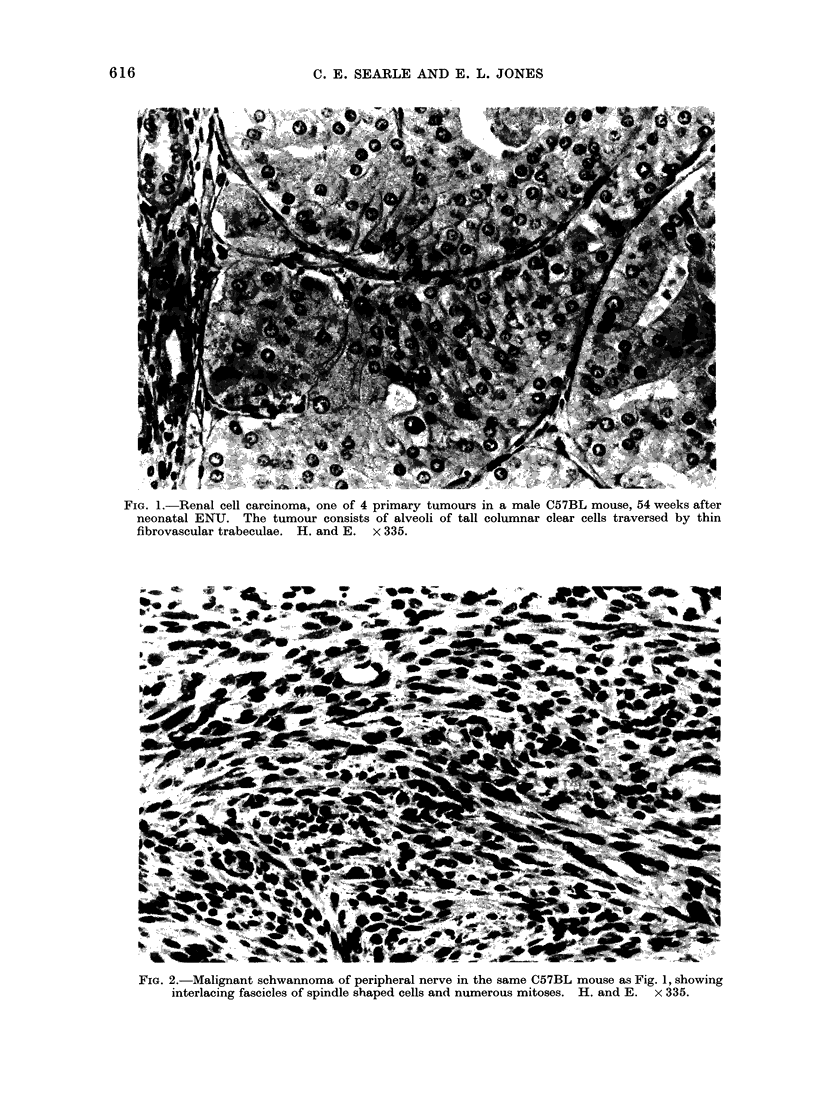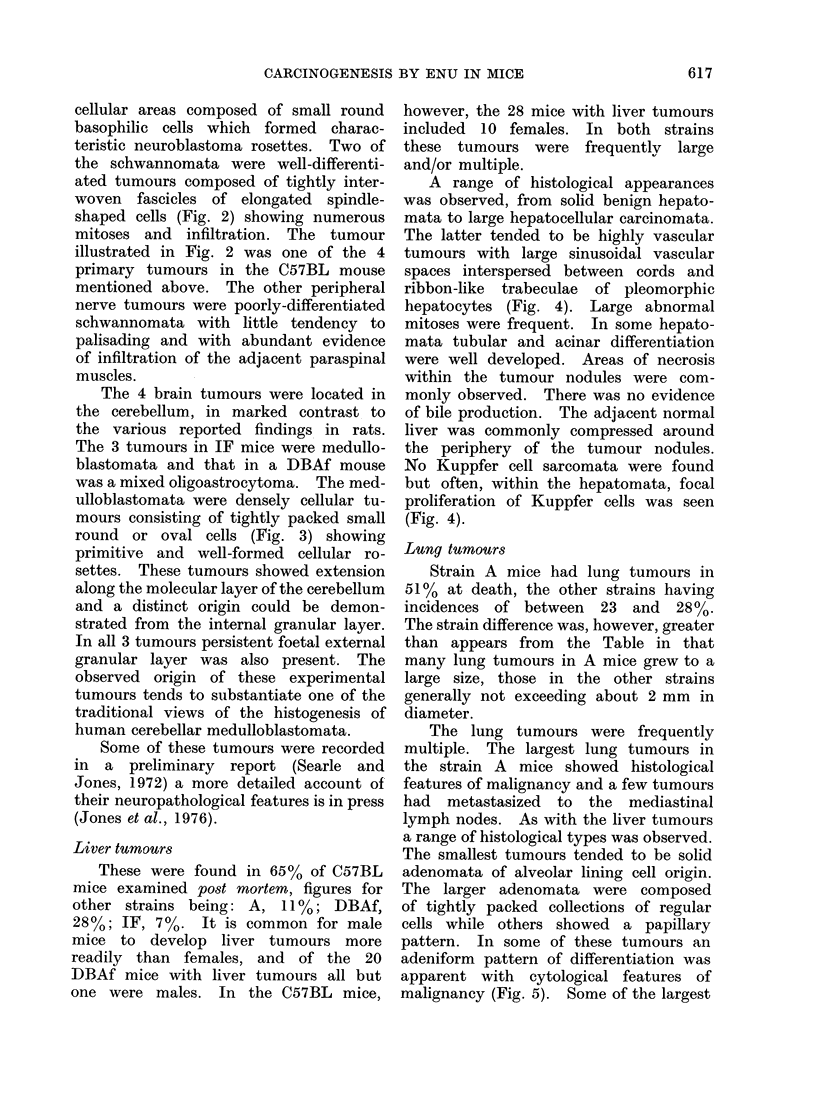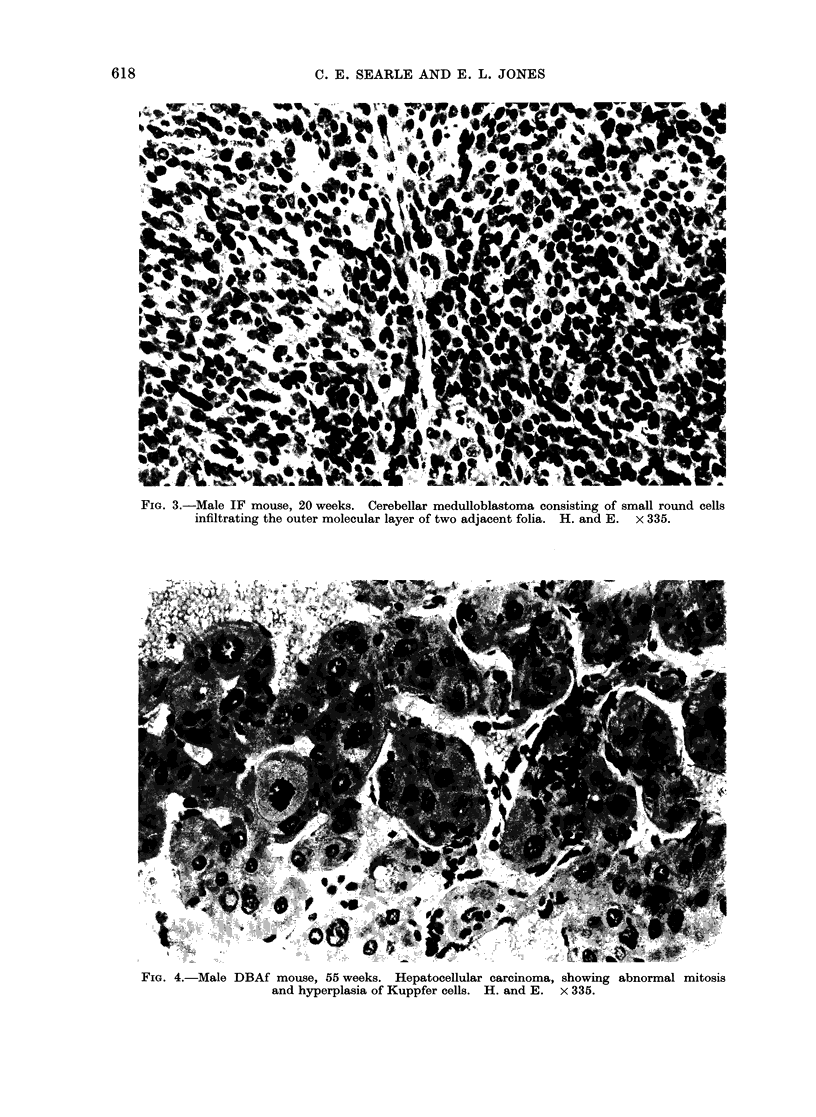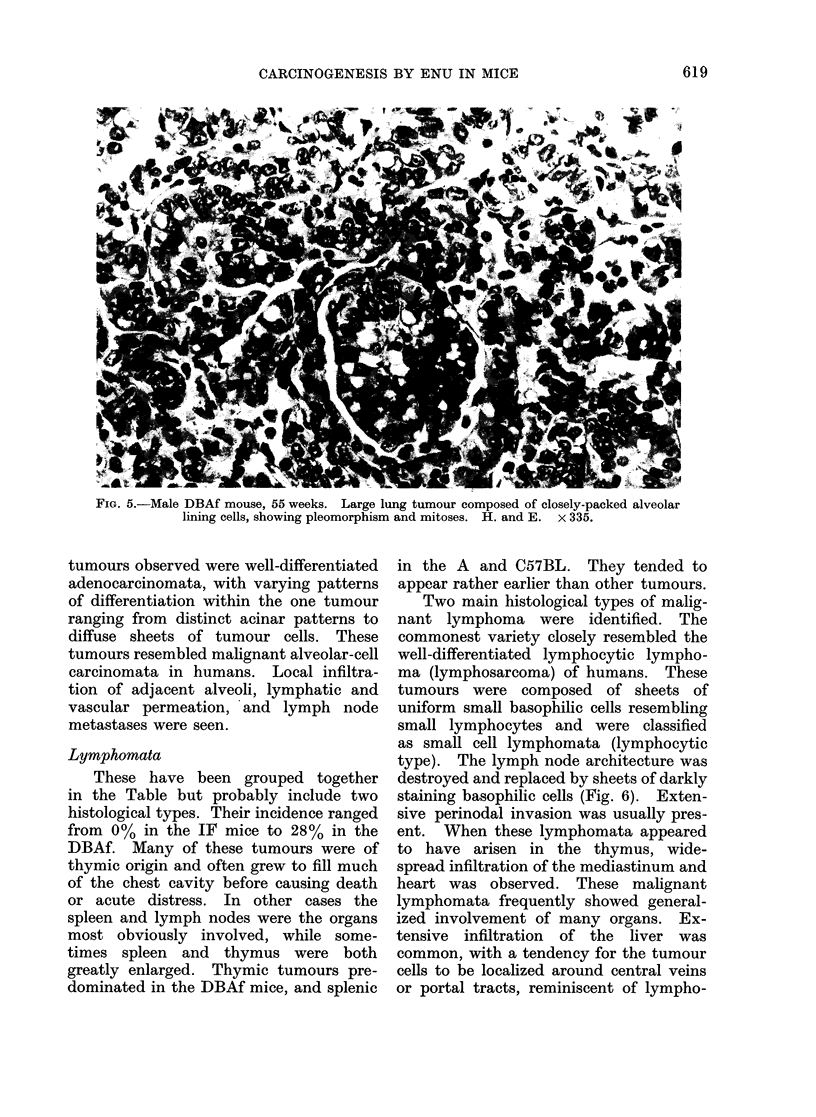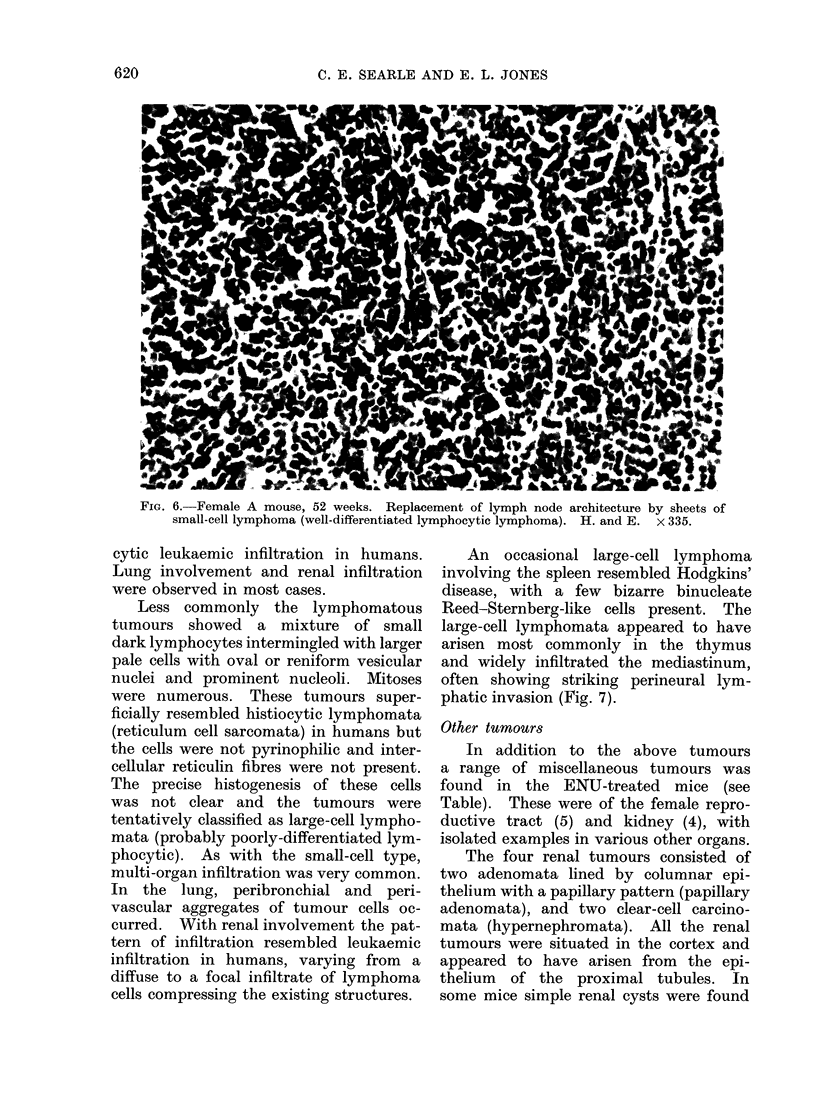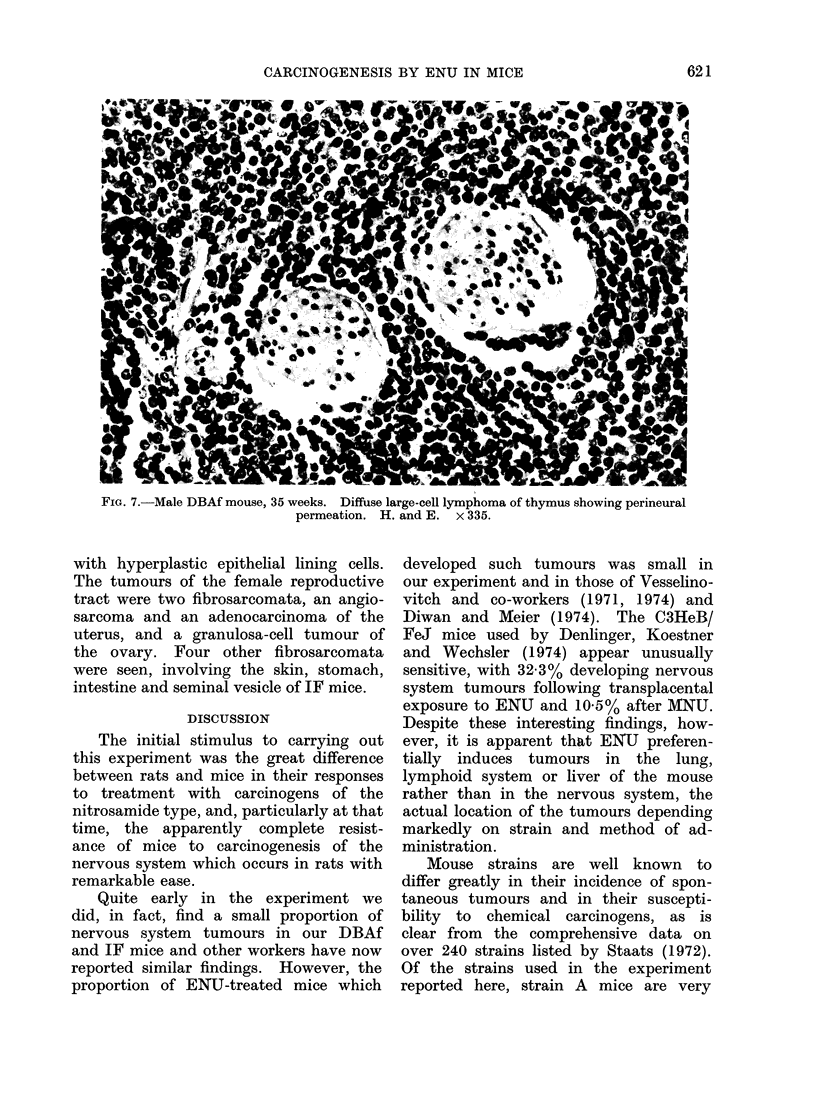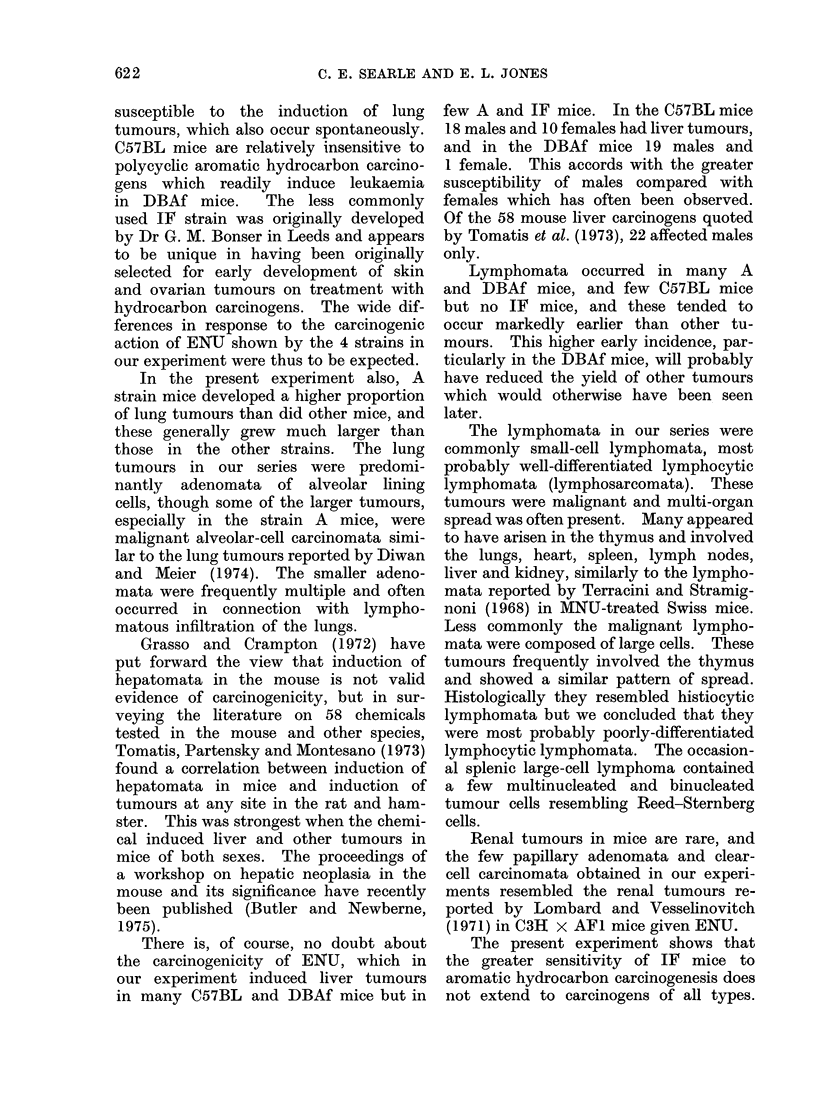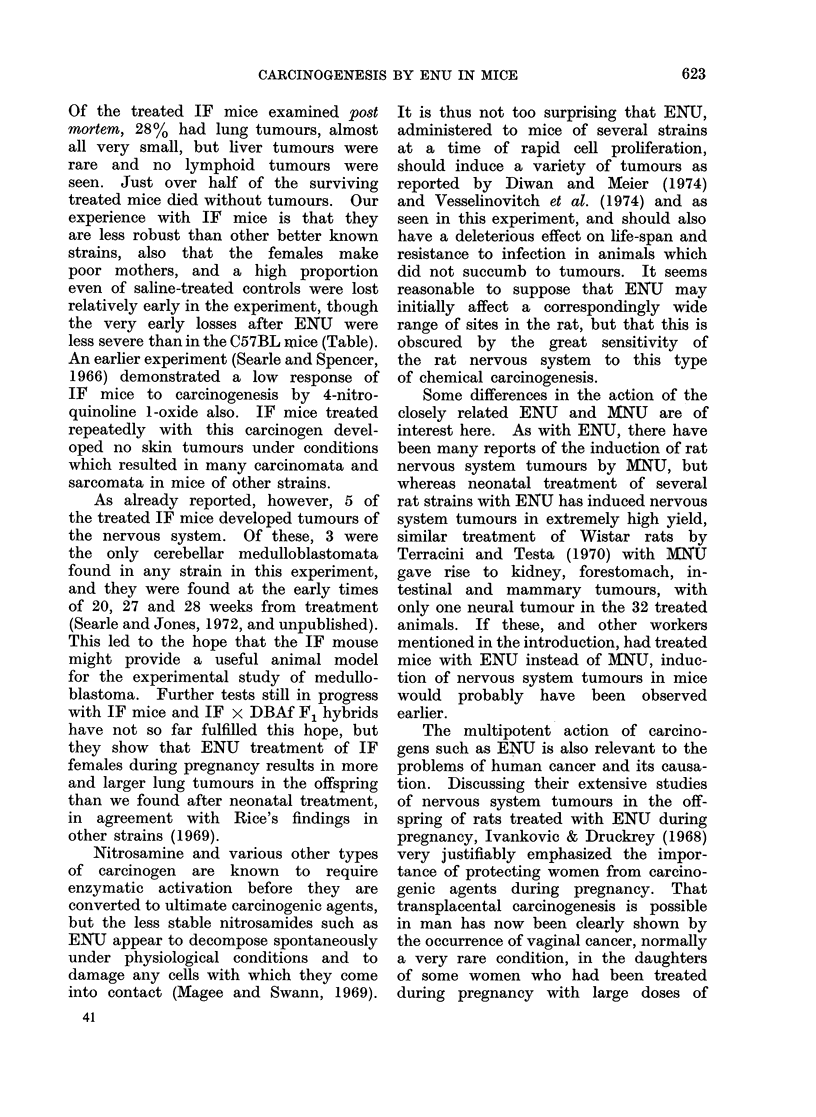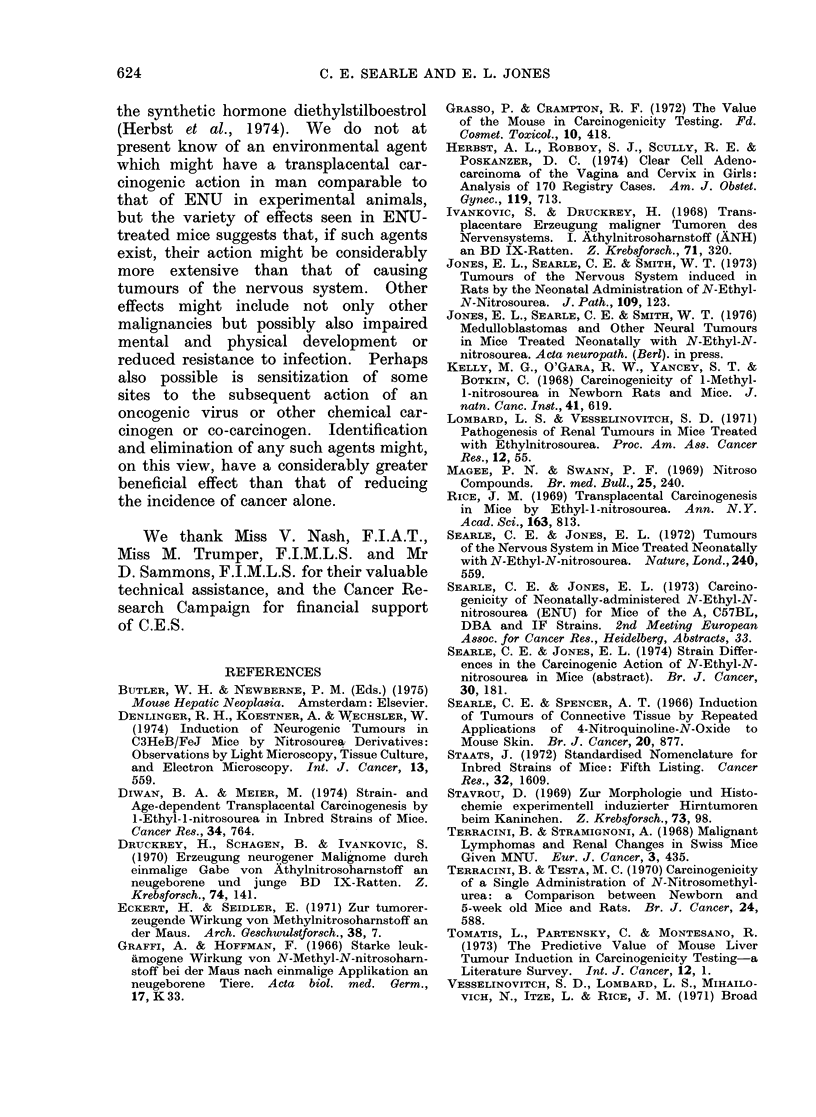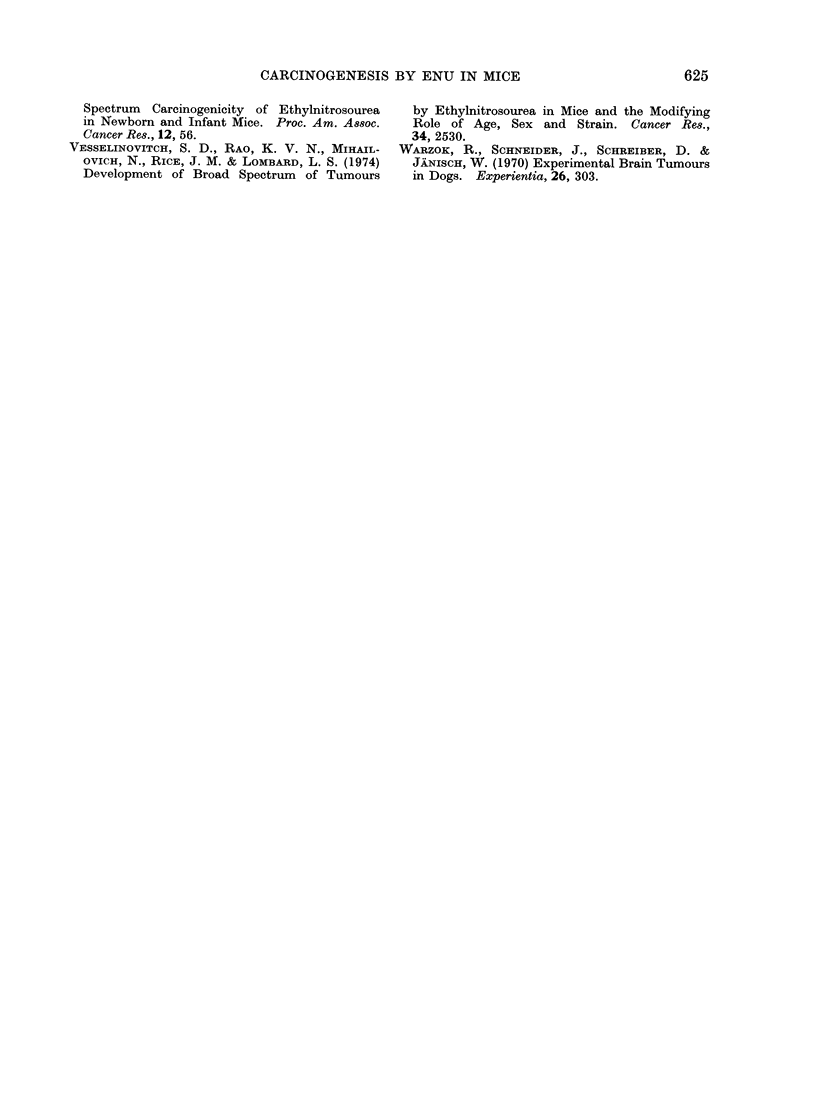# The multipotential carcinogenic action of N-ethyl-N-nitrosourea administered neonatally to mice.

**DOI:** 10.1038/bjc.1976.99

**Published:** 1976-06

**Authors:** C. E. Searle, E. L. Jones

## Abstract

**Images:**


*Br. J. Cancer* (1976) **33**, 612

### THE MULTIPOTENTIAL CARCINOGENIC ACTION OF *N*-ETHYL-*N*-NITROSOUREA ADMINISTERED NEONATALLY TO MICE

C. E. SEARLE AND E. L. JONES

*From the Departments of Cancer Studies and Pathology, University of Birmingham, The Medical School, Birmingham, B*15 2*TJ*

Received 26 November 1975 Accepted 26 January 1976

**Summary.—Newborn A, C57BL, DBAf and IF mice were injected s.c. with a range of doses of *N*-ethyl-*N*-nitrosourea (ENU). A high proportion of treated mice developed tumours, particularly hepatomata, pulmonary adenomata and carcinomata, and malignant lymphomata of thymus and spleen. Liver tumours occurred most frequently in C57BL and DBAf mice, lung tumours in A mice, and lymphomata in A and DBAf mice. A small proportion of C57BL, DBAf and IF mice developed tumours of the nervous system. The results are discussed with reference to the ready induction of nervous system tumours in similarly treated rats, and their relevance for human cancer.**

Carcinogens of the nitrosamide type induce tumours in many organs in experimental animals, but particular interest has been shown in their ability to induce tumours of the central and peripheral nervous systems. The carcinogen which has been extensively used in these experiments has been *N*-ethyl-*N*-nitrosourea (ENU), and as the experimental animal, most studies have used the rat, in which nervous system tumours are induced in up to 100% of animals following exposure to a relatively small single dose of ENU in late foetal life (Ivankovic and Druckrey, 1968) or in the immediate neonatal period (Druckrey, Schagen and Ivankovic, 1970; Jones, Searle and Smith, 1973).

In other species, brain tumours resulted from repeated treatment of the rabbit (Stavrou, 1969) and the dog (Warzok *et al.*, 1970) with *N*-methyl-*N*-nitrosourea (MNU), but results of a number of studies rather surprisingly indicated that nervous system tumours cannot be induced in this way in the mouse. Treatment of mice with MNU has been reported to give rise to leukaemia (Graffi and Hoffmann, 1966), malignant lymphoma (Terracini and Stramignoni, 1968), lymphomata, lung adenomata and hepatomata (Terracini and Testa, 1970) or to benign bronchial adenomata (Eckert and Seidler, 1971). Even when newborn mice were injected intracerebrally with MNU, only leukaemias and lung tumours, but no brain tumours, resulted (Kelly *et al.*, 1968). Similarly, transplacental administration of ENU, which efficiently induces neural tumours in rats, only resulted in lung tumours in mice (Rice, 1969).

We were led by this species difference in response to MNU and ENU to carry out experiments on the effects of ENU administered once neonatally to mice of the 4 inbred strains maintained in the Department of Cancer Studies. We were most interested to find a small number of neural tumours in mice of the DBAf and IF strains, and a brief account of the first 10 mice with such tumours has been published (Searle and Jones, 1972). By the end of the experiment the total had risen to 14, including one C57BL mouse.

However, as in other recently reported studies (Vesselinovitch *et al.*, 1971, 1974; Diwan and Meier, 1974), neural tumours represented only a small proportion of the tumours induced. The principal sites of tumours in the experiment described here were the liver, lung and lymphoid system. Their location depended markedly on strain and many mice had multiple primary tumours. Brief reports were presented earlier (Searle and Jones, 1973, 1974).

### MATERIALS AND METHODS

*N-Ethyl-N-nitrosourea (ENU).*—This was prepared by nitrosation of ethylurea as already described (Jones *et al.*, 1973). As day-old mice weigh only about 1·5 g, however, solutions were prepared for injection containing the desired dose per g body-weight dissolved in 0·02 ml instead of 0·01 ml to enable a slightly larger and more controllable volume to be injected.

*Experimental animals.*—Four strains of mouse were employed for these experiments: A/Bcr, C57BL/Bcr, DBAf/Bcr and IF/Bcr. They were housed on sawdust in plastic cages with tap water available *ad libitum* throughout. They were fed Oxoid breeding diet (Oxo Ltd., London) supplemented by a commercial hamster mix until they were 6 weeks old, after which they received Thompson diet 42 (Heygates Ltd., Bugbrooke Mills, Northants).

Mice were injected s.c. when they were 1 day old with freshly prepared ENU solution using a size 20–25 G 16-mm needle. Mice generally weighed about 1·5 g, the volume administered then being 0·03 ml. The injection solution formed a short-lived blister on the back of the neck, and the young animals were kept apart from their mothers for some 20 min to prevent possible loss of solution through licking. Control mice were treated similarly with saline solution.

A careful watch was kept on the mice, and sick animals or those appearing to bear tumours were killed for examination. Nevertheless, 12 mice were lost to *post mortem* examination through unexpected death and decomposition. Tumours, and other tissues appearing to warrant histological examination, were fixed in FAM (formalin–acetic acid–methanol, 1 : 1 : 8 by volume) and embedded in paraffin wax. Sections (5 μm) were stained routinely with Harris' haematoxylin and eosin and additionally with special stains as required. Because of the particular interest attaching to tumours in the nervous system, brains of all the mice were also processed and examined routinely. With a few exceptions, surviving animals were killed for examination at 80 weeks.

### RESULTS

The Table summarizes our results on the mice of each strain treated neonatally with ENU doses ranging from 0 mg/kg (saline controls) to 160 mg/kg: numbers of mice treated, surviving to 6 weeks, and examined *post mortem*, numbers bearing tumours of various types, and found tumour-free at death. The median number of weeks from treatment to death for each group of mice is shown in parenthesis, and the sign † indicates the presence of additional primary tumours in at least half the animals.

### *Survival of control and treated mice*

Most control mice of the A, C57BL and DBAf strains survived well to the end of the experiment, but many controls in the less robust IF strain died or had to be killed from 45 weeks onwards (see Table).

In the ENU-treated mice, overall survival at 6 weeks was highest in the A strain mice (83·4%) and lowest in the C57BL (42·3%). The proportion of mice which were tumour-free at death (last column of Table) tended to be lowest at intermediate rather than high dosages, but this was due to more mice dying relatively early after the higher dosages. Apart from its tumour-inducing action, the ENU treatment was thus associated also with a shortening of life span, and many treated mice, particularly A and IF, also showed evidence of lung and other infections.

In a further experiment (not shown in the Table) some strain A mice received ENU at 80 mg/kg on each of the 1st,

TABLE.—*Incidence of Tumours in A, C57BL, DBAf and IF Mice Treated Neonatally with Various Doses of ENU*

| Mice | | | | | Mice with tumours | | | | | |
|---|---|---|---|---|---|---|---|---|---|---|
| Strain | Dose (mg/kg) | No. injected | Survivors (6 weeks) | Examined at P.M. | Liver* | Lung* | Lymphoid* | Nervous system* | Others* | Mice tumour-free* |
| A | Control | 15 | 15 | 14 | — | 2 (80) | 1 (71) | — | Adrenocortical carcinoma (80) | 10 (80) |
| | 10 | 20 | 18 | 17 | — | 7 (80) | 4 (53) | — | — | 7 (67) |
| | 20 | 14 | 13 | 13 | 3† (72) | 9† (72) | 3† (68) | — | Kidney adenocarcinoma (73) | 3 (33) |
| | 40 | 17 | 15 | 15 | 4† (69) | 11 (66) | 2 (35) | — | Uterine fibrosarcoma (62) | 2 (39) |
| | 80 | 19 | 17 | 14 | — | 4† (62) | 3† (53) | — | Uterine adenocarcinoma (60) | 8 (37) |
| | 120 | 19 | 17 | 16 | 1† (56) | 7 (55) | 2 (23) | — | Kidney adenomata (43, 55) | 7 (29) |
| | 160 | 7 | 0 | — | — | — | — | — | — | — |
| | All ENU | 96 | 80 | 75 | 8 | 38 | 16 | — | 5 | 27 |
| C57BL | Control | 21 | 15 | 15 | — | — | — | — | Granular cell myoblastoma in hind limb (28) | 14 (80) |
| | 10 | 14 | 7 | 7 | 4 (84) | — | 1 (52) | — | — | 2 (57) |
| | 20 | 17 | 11 | 11 | 7 (78) | 2† (70) | 2† (62) | — | Lacrymal gland tumour (62) | 2 (7) |
| | 40 | 14 | 7 | 7 | 7 (62) | 1† (58) | — | — | — | — |
| | 80 | 21 | 16 | 15 | 8† (56) | 7† (58) | 1† (50) | 1† (54) | Kidney adenocarcinoma (54) | 5 (12) |
| | 120 | 21 | 3 | 3 | 2 (52) | — | 1 (21) | — | — | — |
| | 160 | 13 | 0 | — | — | — | — | — | — | — |
| | All ENU | 100 | 44 | 43 | 28 | 10 | 5 | 1 | 2 | 9 |
| DBAf | Control | 18 | 12 | 12 | — | 1 (80) | — | — | — | 11 (80) |
| | 10 | 11 | 9 | 9 | — | 1 (80) | 1 (41) | 1 (69) | Mammary tumour (69). Uterine angiosarcoma (70) | 4 (79) |
| | 20 | 21 | 20 | 18 | 7 (72) | 6† (78) | 2 (68) | 1 (69) | Uterine fibrosarcoma (72) | 4 (73) |
| | 40 | 28 | 26 | 26 | 9 (68) | 6† (65) | 8 (44) | 3 (53) | — | 4 (45) |
| | 80 | 23 | 10 | 10 | 2† (56) | 2† (48) | 3 (42) | 3† (55) | Adrenocortical carcinoma (70). Skin squamous cell carcinoma (70) | 3 (49) |
| | 120 | 14 | 9 | 8 | 2† (41) | 2† (53) | 5 (31) | — | Ovarian granulosa-cell tumour (37) | — |
| | 160 | 5 | 1 | 1 | — | — | 1 (32) | — | — | — |
| | All ENU | 102 | 75 | 72 | 20 | 17 | 20 | 8 | 6 | 15 |
| IF | Control | 19 | 18 | 17 | — | — | — | — | — | 17 (71) |
| | 10 | 14 | 12 | 12 | — | 1 (79) | — | — | Skin, malignant glandular tumour (72) | 10 (48) |
| | 20 | 9 | 8 | 8 | 1† (68) | 6 (69) | — | — | Intestinal fibrosarcoma (60) | 1 (43) |
| | 40 | 10 | 10 | 8 | 1† (67) | 2† (62) | — | — | Glandular stomach sarcoma (58) | 6 (50) |
| | 80 | 21 | 14 | 14 | 1 (57) | 3 (50) | — | 3 (27) | Skin fibrosarcoma (32). Seminal vesicle sarcoma (23) | 5 (35) |
| | 120 | 17 | 8 | 7 | — | 2 (47) | — | 2 (40) | — | 3 (21) |
| | 160 | 16 | 5 | 3 | 1 (38) | 1 (42) | — | — | — | 3 (42) |
| | All ENU | 87 | 57 | 54 | 4 | 15 | — | 5 | 5 | 28 |

* Median weeks to death in parentheses.

† Additional primary tumours present in 50% or more mice.

2nd and 3rd days after birth. Their 6-week survival was 11/18, but most of the survivors died tumour-free between 16 and 30 weeks. The only tumours found were lymphomata in 2 animals at 18 and 22 weeks and an injection site sarcoma in another at 25 weeks. Though the divided dose was better tolerated than would be expected for a single dose of this magnitude it was thus still too toxic to allow good survival and development of many tumours. For the single neonatal dose technique, the most favourable dose range in the mouse appears to be 20–80 mg/kg.

*Tumours*

A major proportion of the mice treated neonatally with ENU developed tumours. These were predominantly liver, lung and lymphoid tumours but also included a number of tumours in the central and peripheral nervous systems, female reproductive tract, kidney and some other sites (Table). There were 6 mice with tumours among the 58 controls examined *post mortem*.

As is clear from the Table, the proportions of tumours induced at different sites showed marked dependence on strain. Thus, liver tumours occurred predominantly in C57BL and DBAf mice and lymphoid tumours in A and DBAf mice. Though lung tumours occurred in all strains these tended to remain small except in the A mice. With one exception, the nervous system tumours were found only in DBAf and IF mice.

*Multiple primary tumours*

A noticeable feature of this experiment was the high proportion of treated mice which developed primary tumours of more than one type. Additional primary tumours were present in over 40% of mice with liver or lung tumours and in about 15% of those with lymphomata. Liver and lung tumours often occurred together but a variety of other combinations was seen. Where more than half of the mice in a group had two or more primary tumours, this is indicated in the Table.

Of the mice with more than 2 primary tumours, the most interesting was the only C57BL mouse to develop a tumour of the nervous system. In addition to a schwannoma of a peripheral nerve, this animal had primary tumours of the liver and lung and a renal cell carcinoma. The lung tumour consisted of compact small round cells with regular round or oval nuclei. Acinar differentiation was not marked and occasional pleomorphic nuclei and mitoses were found. The tumour was classified as a pulmonary adenoma of alveolar lining cells. The liver tumour was a histologically benign hepatoma. The hepatic architecture was replaced by sheets of large hepatocytes tending to be arranged in cords and trabeculae.

In contrast, the renal and peripheral nervous system tumours were histologically malignant. The renal tumour consisted of tightly packed alveolar or acinar collections of tall columnar clear cells traversed by thin fibrovascular trabeculae (Fig. 1). A moderate number of mitoses were present. Histologically this tumour closely resembled the clear-cell carcinoma of kidney in humans. The peripheral nerve tumour was a malignant schwannoma (Fig. 2) as described below.

*Nervous system tumours*

Fifteen tumours of the nervous system were found in 14 animals (overall incidence 5·74%), all but one of which were DBAf (11·10%) or IF (9·27%) mice. Nine tumours were classified as malignant schwannomata located in the spinal or cranial nerves. One of these tumours originated from the trigeminal ganglion and was a mixed schwannoma and neuroblastoma similar to the trigeminal nerve tumours observed in Wistar-derived rats (Jones *et al.*, 1973). This mixed tumour consisted of interlacing spindle cells, intermixed with densely

FIG. 1.—Renal cell carcinoma, one of 4 primary tumours in a male C57BL mouse, 54 weeks after neonatal ENU. The tumour consists of alveoli of tall columnar clear cells traversed by thin fibrovascular trabeculae. H. and E. ×335.

FIG. 2.—Malignant schwannoma of peripheral nerve in the same C57BL mouse as Fig. 1, showing interlacing fascicles of spindle shaped cells and numerous mitoses. H. and E. ×335.

cellular areas composed of small round basophilic cells which formed characteristic neuroblastoma rosettes. Two of the schwannomata were well-differentiated tumours composed of tightly interwoven fascicles of elongated spindle-shaped cells (Fig. 2) showing numerous mitoses and infiltration. The tumour illustrated in Fig. 2 was one of the 4 primary tumours in the C57BL mouse mentioned above. The other peripheral nerve tumours were poorly-differentiated schwannomata with little tendency to palisading and with abundant evidence of infiltration of the adjacent paraspinal muscles.

The 4 brain tumours were located in the cerebellum, in marked contrast to the various reported findings in rats. The 3 tumours in IF mice were medulloblastomata and that in a DBAf mouse was a mixed oligoastrocytoma. The medulloblastomata were densely cellular tumours consisting of tightly packed small round or oval cells (Fig. 3) showing primitive and well-formed cellular rosettes. These tumours showed extension along the molecular layer of the cerebellum and a distinct origin could be demonstrated from the internal granular layer. In all 3 tumours persistent foetal external granular layer was also present. The observed origin of these experimental tumours tends to substantiate one of the traditional views of the histogenesis of human cerebellar medulloblastomata.

Some of these tumours were recorded in a preliminary report (Searle and Jones, 1972) a more detailed account of their neuropathological features is in press (Jones *et al.*, 1976).

*Liver tumours*

These were found in 65% of C57BL mice examined *post mortem*, figures for other strains being: A, 11%; DBAf, 28%; IF, 7%. It is common for male mice to develop liver tumours more readily than females, and of the 20 DBAf mice with liver tumours all but one were males. In the C57BL mice, however, the 28 mice with liver tumours included 10 females. In both strains these tumours were frequently large and/or multiple.

A range of histological appearances was observed, from solid benign hepatomata to large hepatocellular carcinomata. The latter tended to be highly vascular tumours with large sinusoidal vascular spaces interspersed between cords and ribbon-like trabeculae of pleomorphic hepatocytes (Fig. 4). Large abnormal mitoses were frequent. In some hepatomata tubular and acinar differentiation were well developed. Areas of necrosis within the tumour nodules were commonly observed. There was no evidence of bile production. The adjacent normal liver was commonly compressed around the periphery of the tumour nodules. No Kuppfer cell sarcomata were found but often, within the hepatomata, focal proliferation of Kuppfer cells was seen (Fig. 4).

*Lung tumours*

Strain A mice had lung tumours in 51% at death, the other strains having incidences of between 23 and 28%. The strain difference was, however, greater than appears from the Table in that many lung tumours in A mice grew to a large size, those in the other strains generally not exceeding about 2 mm in diameter.

The lung tumours were frequently multiple. The largest lung tumours in the strain A mice showed histological features of malignancy and a few tumours had metastasized to the mediastinal lymph nodes. As with the liver tumours a range of histological types was observed. The smallest tumours tended to be solid adenomata of alveolar lining cell origin. The larger adenomata were composed of tightly packed collections of regular cells while others showed a papillary pattern. In some of these tumours an adeniform pattern of differentiation was apparent with cytological features of malignancy (Fig. 5). Some of the largest

FIG. 3.—Male IF mouse, 20 weeks. Cerebellar medulloblastoma consisting of small round cells infiltrating the outer molecular layer of two adjacent folia. H. and E. ×335.

FIG. 4.—Male DBAf mouse, 55 weeks. Hepatocellular carcinoma, showing abnormal mitosis and hyperplasia of Kuppfer cells. H. and E. ×335.

FIG. 5.—Male DBAf mouse, 55 weeks. Large lung tumour composed of closely-packed alveolar lining cells, showing pleomorphism and mitoses. H. and E. ×335.

tumours observed were well-differentiated adenocarcinomata, with varying patterns of differentiation within the one tumour ranging from distinct acinar patterns to diffuse sheets of tumour cells. These tumours resembled malignant alveolar-cell carcinomata in humans. Local infiltration of adjacent alveoli, lymphatic and vascular permeation, and lymph node metastases were seen.

*Lymphomata*

These have been grouped together in the Table but probably include two histological types. Their incidence ranged from 0% in the IF mice to 28% in the DBAf. Many of these tumours were of thymic origin and often grew to fill much of the chest cavity before causing death or acute distress. In other cases the spleen and lymph nodes were the organs most obviously involved, while sometimes spleen and thymus were both greatly enlarged. Thymic tumours predominated in the DBAf mice, and splenic in the A and C57BL. They tended to appear rather earlier than other tumours.

Two main histological types of malignant lymphoma were identified. The commonest variety closely resembled the well-differentiated lymphocytic lymphoma (lymphosarcoma) of humans. These tumours were composed of sheets of uniform small basophilic cells resembling small lymphocytes and were classified as small cell lymphomata (lymphocytic type). The lymph node architecture was destroyed and replaced by sheets of darkly staining basophilic cells (Fig. 6). Extensive perinodal invasion was usually present. When these lymphomata appeared to have arisen in the thymus, widespread infiltration of the mediastinum and heart was observed. These malignant lymphomata frequently showed generalized involvement of many organs. Extensive infiltration of the liver was common, with a tendency for the tumour cells to be localized around central veins or portal tracts, reminiscent of lympho-

FIG. 6.—Female A mouse, 52 weeks. Replacement of lymph node architecture by sheets of small-cell lymphoma (well-differentiated lymphocytic lymphoma). H. and E. ×335.

cytic leukaemic infiltration in humans. Lung involvement and renal infiltration were observed in most cases.

Less commonly the lymphomatous tumours showed a mixture of small dark lymphocytes intermingled with larger pale cells with oval or reniform vesicular nuclei and prominent nucleoli. Mitoses were numerous. These tumours superficially resembled histiocytic lymphomata (reticulum cell sarcomata) in humans but the cells were not pyrinophilic and intercellular reticulin fibres were not present. The precise histogenesis of these cells was not clear and the tumours were tentatively classified as large-cell lymphomata (probably poorly-differentiated lymphocytic). As with the small-cell type, multi-organ infiltration was very common. In the lung, peribronchial and perivascular aggregates of tumour cells occurred. With renal involvement the pattern of infiltration resembled leukaemic infiltration in humans, varying from a diffuse to a focal infiltrate of lymphoma cells compressing the existing structures.

An occasional large-cell lymphoma involving the spleen resembled Hodgkins' disease, with a few bizarre binucleate Reed–Sternberg-like cells present. The large-cell lymphomata appeared to have arisen most commonly in the thymus and widely infiltrated the mediastinum, often showing striking perineural lymphatic invasion (Fig. 7).

*Other tumours*

In addition to the above tumours a range of miscellaneous tumours was found in the ENU-treated mice (see Table). These were of the female reproductive tract (5) and kidney (4), with isolated examples in various other organs.

The four renal tumours consisted of two adenomata lined by columnar epithelium with a papillary pattern (papillary adenomata), and two clear-cell carcinomata (hypernephromata). All the renal tumours were situated in the cortex and appeared to have arisen from the epithelium of the proximal tubules. In some mice simple renal cysts were found

FIG. 7.—Male DBAf mouse, 35 weeks. Diffuse large-cell lymphoma of thymus showing perineural permeation. H. and E. × 335.

with hyperplastic epithelial lining cells. The tumours of the female reproductive tract were two fibrosarcomata, an angiosarcoma and an adenocarcinoma of the uterus, and a granulosa-cell tumour of the ovary. Four other fibrosarcomata were seen, involving the skin, stomach, intestine and seminal vesicle of IF mice.

### DISCUSSION

The initial stimulus to carrying out this experiment was the great difference between rats and mice in their responses to treatment with carcinogens of the nitrosamide type, and, particularly at that time, the apparently complete resistance of mice to carcinogenesis of the nervous system which occurs in rats with remarkable ease.

Quite early in the experiment we did, in fact, find a small proportion of nervous system tumours in our DBAf and IF mice and other workers have now reported similar findings. However, the proportion of ENU-treated mice which developed such tumours was small in our experiment and in those of Vesselinovitch and co-workers (1971, 1974) and Diwan and Meier (1974). The C3HeB/FeJ mice used by Denlinger, Koestner and Wechsler (1974) appear unusually sensitive, with 32·3% developing nervous system tumours following transplacental exposure to ENU and 10·5% after MNU. Despite these interesting findings, however, it is apparent that ENU preferentially induces tumours in the lung, lymphoid system or liver of the mouse rather than in the nervous system, the actual location of the tumours depending markedly on strain and method of administration.

Mouse strains are well known to differ greatly in their incidence of spontaneous tumours and in their susceptibility to chemical carcinogens, as is clear from the comprehensive data on over 240 strains listed by Staats (1972). Of the strains used in the experiment reported here, strain A mice are very susceptible to the induction of lung tumours, which also occur spontaneously. C57BL mice are relatively insensitive to polycyclic aromatic hydrocarbon carcinogens which readily induce leukaemia in DBAf mice. The less commonly used IF strain was originally developed by Dr G. M. Bonser in Leeds and appears to be unique in having been originally selected for early development of skin and ovarian tumours on treatment with hydrocarbon carcinogens. The wide differences in response to the carcinogenic action of ENU shown by the 4 strains in our experiment were thus to be expected.

In the present experiment also, A strain mice developed a higher proportion of lung tumours than did other mice, and these generally grew much larger than those in the other strains. The lung tumours in our series were predominantly adenomata of alveolar lining cells, though some of the larger tumours, especially in the strain A mice, were malignant alveolar-cell carcinomata similar to the lung tumours reported by Diwan and Meier (1974). The smaller adenomata were frequently multiple and often occurred in connection with lymphomatous infiltration of the lungs.

Grasso and Crampton (1972) have put forward the view that induction of hepatomata in the mouse is not valid evidence of carcinogenicity, but in surveying the literature on 58 chemicals tested in the mouse and other species, Tomatis, Partensky and Montesano (1973) found a correlation between induction of hepatomata in mice and induction of tumours at any site in the rat and hamster. This was strongest when the chemical induced liver and other tumours in mice of both sexes. The proceedings of a workshop on hepatic neoplasia in the mouse and its significance have recently been published (Butler and Newberne, 1975).

There is, of course, no doubt about the carcinogenicity of ENU, which in our experiment induced liver tumours in many C57BL and DBAf mice but in few A and IF mice. In the C57BL mice 18 males and 10 females had liver tumours, and in the DBAf mice 19 males and 1 female. This accords with the greater susceptibility of males compared with females which has often been observed. Of the 58 mouse liver carcinogens quoted by Tomatis *et al.* (1973), 22 affected males only.

Lymphomata occurred in many A and DBAf mice, and few C57BL mice but no IF mice, and these tended to occur markedly earlier than other tumours. This higher early incidence, particularly in the DBAf mice, will probably have reduced the yield of other tumours which would otherwise have been seen later.

The lymphomata in our series were commonly small-cell lymphomata, most probably well-differentiated lymphocytic lymphomata (lymphosarcomata). These tumours were malignant and multi-organ spread was often present. Many appeared to have arisen in the thymus and involved the lungs, heart, spleen, lymph nodes, liver and kidney, similarly to the lymphomata reported by Terracini and Stramignoni (1968) in MNU-treated Swiss mice. Less commonly the malignant lymphomata were composed of large cells. These tumours frequently involved the thymus and showed a similar pattern of spread. Histologically they resembled histiocytic lymphomata but we concluded that they were most probably poorly-differentiated lymphocytic lymphomata. The occasional splenic large-cell lymphoma contained a few multinucleated and binucleated tumour cells resembling Reed–Sternberg cells.

Renal tumours in mice are rare, and the few papillary adenomata and clear-cell carcinomata obtained in our experiments resembled the renal tumours reported by Lombard and Vesselinovitch (1971) in C3H $\times$ AF1 mice given ENU.

The present experiment shows that the greater sensitivity of IF mice to aromatic hydrocarbon carcinogenesis does not extend to carcinogens of all types.

Of the treated IF mice examined *post mortem*, 28% had lung tumours, almost all very small, but liver tumours were rare and no lymphoid tumours were seen. Just over half of the surviving treated mice died without tumours. Our experience with IF mice is that they are less robust than other better known strains, also that the females make poor mothers, and a high proportion even of saline-treated controls were lost relatively early in the experiment, though the very early losses after ENU were less severe than in the C57BL mice (Table). An earlier experiment (Searle and Spencer, 1966) demonstrated a low response of IF mice to carcinogenesis by 4-nitroquinoline 1-oxide also. IF mice treated repeatedly with this carcinogen developed no skin tumours under conditions which resulted in many carcinomata and sarcomata in mice of other strains.

As already reported, however, 5 of the treated IF mice developed tumours of the nervous system. Of these, 3 were the only cerebellar medulloblastomata found in any strain in this experiment, and they were found at the early times of 20, 27 and 28 weeks from treatment (Searle and Jones, 1972, and unpublished). This led to the hope that the IF mouse might provide a useful animal model for the experimental study of medulloblastoma. Further tests still in progress with IF mice and IF $\times$ DBAf $F_1$ hybrids have not so far fulfilled this hope, but they show that ENU treatment of IF females during pregnancy results in more and larger lung tumours in the offspring than we found after neonatal treatment, in agreement with Rice's findings in other strains (1969).

Nitrosamine and various other types of carcinogen are known to require enzymatic activation before they are converted to ultimate carcinogenic agents, but the less stable nitrosamides such as ENU appear to decompose spontaneously under physiological conditions and to damage any cells with which they come into contact (Magee and Swann, 1969). It is thus not too surprising that ENU, administered to mice of several strains at a time of rapid cell proliferation, should induce a variety of tumours as reported by Diwan and Meier (1974) and Vesselinovitch *et al.* (1974) and as seen in this experiment, and should also have a deleterious effect on life-span and resistance to infection in animals which did not succumb to tumours. It seems reasonable to suppose that ENU may initially affect a correspondingly wide range of sites in the rat, but that this is obscured by the great sensitivity of the rat nervous system to this type of chemical carcinogenesis.

Some differences in the action of the closely related ENU and MNU are of interest here. As with ENU, there have been many reports of the induction of rat nervous system tumours by MNU, but whereas neonatal treatment of several rat strains with ENU has induced nervous system tumours in extremely high yield, similar treatment of Wistar rats by Terracini and Testa (1970) with MNU gave rise to kidney, forestomach, intestinal and mammary tumours, with only one neural tumour in the 32 treated animals. If these, and other workers mentioned in the introduction, had treated mice with ENU instead of MNU, induction of nervous system tumours in mice would probably have been observed earlier.

The multipotent action of carcinogens such as ENU is also relevant to the problems of human cancer and its causation. Discussing their extensive studies of nervous system tumours in the offspring of rats treated with ENU during pregnancy, Ivankovic & Druckrey (1968) very justifiably emphasized the importance of protecting women from carcinogenic agents during pregnancy. That transplacental carcinogenesis is possible in man has now been clearly shown by the occurrence of vaginal cancer, normally a very rare condition, in the daughters of some women who had been treated during pregnancy with large doses of the synthetic hormone diethylstilboestrol (Herbst *et al.*, 1974). We do not at present know of an environmental agent which might have a transplacental carcinogenic action in man comparable to that of ENU in experimental animals, but the variety of effects seen in ENU-treated mice suggests that, if such agents exist, their action might be considerably more extensive than that of causing tumours of the nervous system. Other effects might include not only other malignancies but possibly also impaired mental and physical development or reduced resistance to infection. Perhaps also possible is sensitization of some sites to the subsequent action of an oncogenic virus or other chemical carcinogen or co-carcinogen. Identification and elimination of any such agents might, on this view, have a considerably greater beneficial effect than that of reducing the incidence of cancer alone.

We thank Miss V. Nash, F.I.A.T., Miss M. Trumper, F.I.M.L.S. and Mr D. Sammons, F.I.M.L.S. for their valuable technical assistance, and the Cancer Research Campaign for financial support of C.E.S.

### REFERENCES

Butler, W. H. & Newberne, P. M. (Eds.) (1975) *Mouse Hepatic Neoplasia.* Amsterdam: Elsevier.

Denlinger, R. H., Koestner, A. & Wechsler, W. (1974) Induction of Neurogenic Tumours in C3HeB/FeJ Mice by Nitrosourea Derivatives: Observations by Light Microscopy, Tissue Culture, and Electron Microscopy. *Int. J. Cancer*, **13**, 559.

Diwan, B. A. & Meier, M. (1974) Strain- and Age-dependent Transplacental Carcinogenesis by 1-Ethyl-1-nitrosourea in Inbred Strains of Mice. *Cancer Res.*, **34**, 764.

Druckrey, H., Schagen, B. & Ivankovic, S. (1970) Erzeugung neurogener Malignome durch einmalige Gabe von Äthylnitrosoharnstoff an neugeborene und junge BD IX-Ratten. *Z. Krebsforsch.*, **74**, 141.

Eckert, H. & Seidler, E. (1971) Zur tumorerzeugende Wirkung von Methylnitrosoharnstoff an der Maus. *Arch. Geschwulstforsch.*, **38**, 7.

Graffi, A. & Hoffman, F. (1966) Starke leukämogene Wirkung von *N*-Methyl-*N*-nitrosoharnstoff bei der Maus nach einmalige Applikation an neugeborene Tiere. *Acta biol. med. Germ.*, **17**, K 33.

Grasso, P. & Crampton, R. F. (1972) The Value of the Mouse in Carcinogenicity Testing. *Fd. Cosmet. Toxicol.*, **10**, 418.

Herbst, A. L., Robboy, S. J., Scully, R. E. & Poskanzer, D. C. (1974) Clear Cell Adenocarcinoma of the Vagina and Cervix in Girls: Analysis of 170 Registry Cases. *Am. J. Obstet. Gynec.*, **119**, 713.

Ivankovic, S. & Druckrey, H. (1968) Transplacentare Erzeugung maligner Tumoren des Nervensystems. I. Äthylnitrosoharnstoff (ÄNH) an BD IX-Ratten. *Z. Krebsforsch.*, **71**, 320.

Jones, E. L., Searle, C. E. & Smith, W. T. (1973) Tumours of the Nervous System induced in Rats by the Neonatal Administration of *N*-Ethyl-*N*-Nitrosourea. *J. Path.*, **109**, 123.

Jones, E. L., Searle, C. E. & Smith, W. T. (1976) Medulloblastomas and Other Neural Tumours in Mice Treated Neonatally with *N*-Ethyl-*N*-nitrosourea. *Acta neuropath. (Berl).* in press.

Kelly, M. G., O'Gara, R. W., Yancey, S. T. & Botkin, C. (1968) Carcinogenicity of 1-Methyl-1-nitrosourea in Newborn Rats and Mice. *J. natn. Canc. Inst.*, **41**, 619.

Lombard, L. S. & Vesselinovitch, S. D. (1971) Pathogenesis of Renal Tumours in Mice Treated with Ethylnitrosourea. *Proc. Am. Ass. Cancer Res.*, **12**, 55.

Magee, P. N. & Swann, P. F. (1969) Nitroso Compounds. *Br. med. Bull.*, **25**, 240.

Rice, J. M. (1969) Transplacental Carcinogenesis in Mice by Ethyl-1-nitrosourea. *Ann. N.Y. Acad. Sci.*, **163**, 813.

Searle, C. E. & Jones, E. L. (1972) Tumours of the Nervous System in Mice Treated Neonatally with *N*-Ethyl-*N*-nitrosourea. *Nature, Lond.*, **240**, 559.

Searle, C. E. & Jones, E. L. (1973) Carcinogenicity of Neonatally-administered *N*-Ethyl-*N*-nitrosourea (ENU) for Mice of the A, C57BL, DBA and IF Strains. *2nd Meeting European Assoc. for Cancer Res., Heidelberg, Abstracts*, *33*.

Searle, C. E. & Jones, E. L. (1974) Strain Differences in the Carcinogenic Action of *N*-Ethyl-*N*-nitrosourea in Mice (abstract). *Br. J. Cancer*, **30**, 181.

Searle, C. E. & Spencer, A. T. (1966) Induction of Tumours of Connective Tissue by Repeated Applications of 4-Nitroquinoline-*N*-Oxide to Mouse Skin. *Br. J. Cancer*, **20**, 877.

Staats, J. (1972) Standardised Nomenclature for Inbred Strains of Mice: Fifth Listing. *Cancer Res.*, **32**, 1609.

Stavrou, D. (1969) Zur Morphologie und Histochemie experimentell induzierter Hirntumoren beim Kaninchen. *Z. Krebsforsch.*, **73**, 98.

Terracini, B. & Stramignoni, A. (1968) Malignant Lymphomas and Renal Changes in Swiss Mice Given MNU. *Eur. J. Cancer*, **3**, 435.

Terracini, B. & Testa, M. C. (1970) Carcinogenicity of a Single Administration of *N*-Nitrosomethylurea: a Comparison between Newborn and 5-week old Mice and Rats. *Br. J. Cancer*, **24**, 588.

Tomatis, L., Partensky, C. & Montesano, R. (1973) The Predictive Value of Mouse Liver Tumour Induction in Carcinogenicity Testing—a Literature Survey. *Int. J. Cancer*, **12**, 1.

Vesselinovitch, S. D., Lombard, L. S., Mihailovich, N., Itze, L. & Rice, J. M. (1971) Broad

Spectrum Carcinogenicity of Ethylnitrosourea in Newborn and Infant Mice. *Proc. Am. Assoc. Cancer Res.*, **12**, 56.

VESSELINOVITCH, S. D., RAO, K. V. N., MIHAILOVICH, N., RICE, J. M. & LOMBARD, L. S. (1974) Development of Broad Spectrum of Tumours by Ethylnitrosourea in Mice and the Modifying Role of Age, Sex and Strain. *Cancer Res.*, **34**, 2530.

WARZOK, R., SCHNEIDER, J., SCHREIBER, D. & JÄNISCH, W. (1970) Experimental Brain Tumours in Dogs. *Experientia*, **26**, 303.